# Human and animal epidemic of Yersinia enterocolitica O:9, 1989-1997, Auvergne, France.

**DOI:** 10.3201/eid0505.990516

**Published:** 1999

**Authors:** F Gourdon, J Beytout, A Reynaud, J P Romaszko, D Perre, P Theodore, H Soubelet, J Sirot

**Affiliations:** University Hospital of Clermont-Ferrand, Clermont-Ferrand, France. fgourdon@chu-clermontferrand.fr

## Abstract

Yersinia enterocolitica O:9 infections were reported in Auvergne in 1988 to 1989, while brucellosis due to Brucella abortus was almost eliminated. The serologic cross-reactions between the two bacteria complicated the diagnosis of brucellosis cases. In 1996, human cases of Yersinia enterocolitica O:9 infection were detected, with a peak incidence of 12 cases. Veterinary surveillance could have predicted the emergence of this disease in humans.


### Human and Animal Epidemic of *Yersinia enterocolitica* O:9, 1989–1997, Auvergne, France

**Florence Gourdon,* Jean Beytout,* Alain Reynaud,† Jean-Pierre Romaszko,* Didier Perre,‡ Philippe Theodore,‡ Hélène Soubelet,‡ and Jacques Sirot***
*University Hospital of Clermont-Ferrand, Clermont-Ferrand, France;
†Laboratoire Départemental d'Analyses Vétérinaires, Lempdes, France;
‡Direction Départementale de l'Agriculture et de la Forêt,
Préfecture du Puy-de-Dôme, Lempdes, France

*Yersinia enterocolitica* O:9 infections were reported in Auvergne in 1988 to 1989, while brucellosis due to *Brucella abortus* was almost eliminated. The serologic cross-reactions between the two bacteria complicated the diagnosis of brucellosis cases. In 1996, human cases of *Yersinia enterocolitica* O:9 infection were detected, with a peak incidence of 12 cases. Veterinary surveillance could have predicted the emergence of this disease in humans.

In Auvergne, a cattle-raising area in central France, brucellosis control measures have been strictly observed since 1965, and systematic vaccination was stopped in 1983. Active surveillance is conducted on the basis of clinical findings (abortions or orchitis) and an annual serologic test performed for every animal (rose bengal plate agglutination test or complement fixation test); abortions and orchitis have to be bacteriologically confirmed. When infected animals are detected, a second test on a new sample drawn 2 weeks later is required for confirmation. When an animal on a farm is infected, the herd is slaughtered. This policy has resulted in a dramatic decrease in the prevalence of brucellosis, and very few cases were reported in 1988 (1). In 1988, however, several animals had positive tests for brucellosis. These positive reactions apparently were associated with an epizootic due to *Yersinia enterocolitica* O:9. The bacterium was isolated from the stools of cattle and goats in infected herds (2).

*Yersinia enterocolitica* O:9 shares antigens with *Brucella abortus,* and misdiagnosis can occur because both bacteria produce positive reactions with the Wright agglutination test and immunofluorescent assay (brucellosis) and the agglutination test (yersiniosis) (3). The clinical, biological, and epidemiologic features of the two diseases, however, are quite different. In the 1988 epizootic, *Yersinia* infection, commonly called "atypical brucellosis," affected a few young cattle (<2 years of age) and did not spread to the whole herd. No increase in abortion was noted, the titer of antibodies declined rapidly, and no reaction to the Brucellallergene (Rhone Merieux OCB) dermal test was observed. Epidemiologic and serologic surveillance of infected herds found no evidence of brucellosis infection and allowed restoring them to noninfected status after several months. Epidemiologic surveillance demonstrated that brucellosis decreased, whereas yersiniosis continued to spread throughout the region in the 1990s (Figure 1).

Before the 1988 epizootic, *Y. enterocolitica* human infections were rare in Auvergne. In a 1980-81 survey of infections due to *Yersinia* species, five patients had antibodies against *Y. enterocolitica* O:3, the serogroup commonly found in Europe in this period (4,5). No more than three cases of *Y. enterocolitica* infection were recorded each year at the University Hospital laboratory during 1982 to 1990: none had the serotype O:9. Two cases of human

Corresponding author: Florence Gourdon, University Hospital of Clermont-Ferrand, Hôtel Dieu, Boulevard Leon Malfreyt, 63000 Clermont-Ferrand, France; fax: 33-473-316-264; e-mail: fgourdon@chu-clermontferrand.fr.

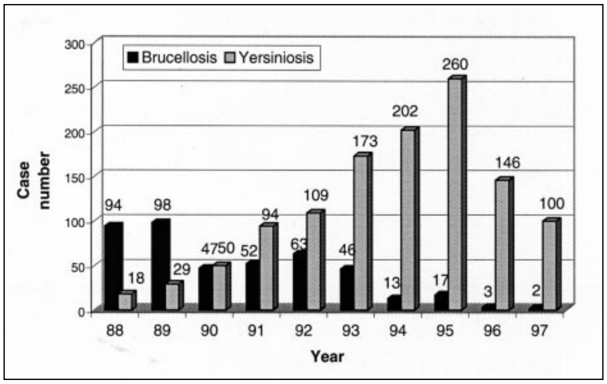

Figure 1. Annual incidence of cattle brucellosis and yersiniosis, Auvergne, France, 1989–1997.

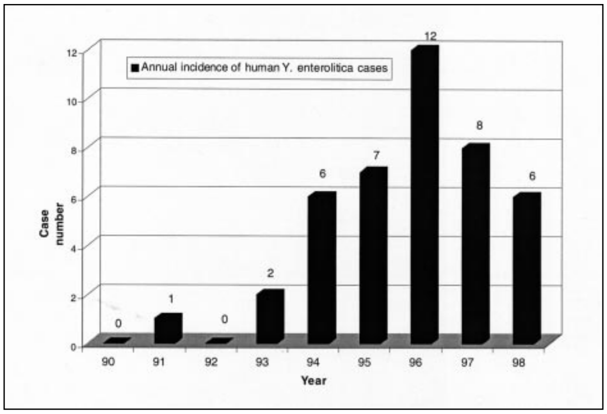

Figure 2. *Yersinia enterocolitica* O:9 infections in humans, Auvergne, France, 1990–1998.

autochthonous brucellosis were detected in 1988 to 1990; in both cases, brucellosis had been detected in the patients' cattle a few months before.

Awareness of *Yersinia* infection was heightened in the regional teaching hospital, but systematic surveillance for patients with diarrhea or abdominal symptoms could not be established. The first human case was detected in 1991; this patient also had positive serologic results for brucellosis but no history of contact with *Brucella*-infected animals; gastrointestinal symptoms suggested yersiniosis (5). Since then, the number of human cases diagnosed in Auvergne has increased, despite the lack of systematic screening for *Yersinia* infection. Human yersiniosis cases were defined by clinical symptoms (fever, gastrointestinal symptoms, arthritis, *erythema nodosum*) associated with a positive serologic test for brucellosis and lack of contact with *Brucella*-infected animals.

In 1996, a retrospective study was done among regional medical laboratories to identify positive brucellosis serologic tests from April 1995 to March 1996. Of eight cases detected, six met criteria for yersiniosis and two had evidence of past brucellosis. Through the end of 1998, 42 cases were recorded, with a peak incidence of 12 cases in 1996 (Figure 2). Gastrointestinal symptoms were found in 35 (83%) patients: diarrhea alone in eight, abdominal pain in six (four patients had surgery [6]), and both in 21. Twelve patients had fever with no other symptoms when they sought medical attention (7), six had arthritis in one or several joints (two with sacroilieitis), and five had *erythema nodosum*. The diagnosis of the last 18 cases was confirmed with an enzyme-linked immunosor bent assay (ELISA) developed and performed by the Laboratoire de Reference des Yersinia of Institut Pasteur. This new ELISA, which uses microtitration plates coated with plasmid-encoded *Yersinia* outer proteins (YOP), is more specific than the agglutination test (8). The results matched the clinical diagnosis of yersiniosis and were consistently negative in patients with brucellosis (six recent or past cases with a positive Wright agglutination test) tested in the same period. Stool samples were negative except for one, but in most cases, gastrointestinal symptoms disappeared before the patients were admitted to the hospital. None of these patients had had contact with *Brucella*-infected animals. Only six were cattle breeders, and seven had recent contact with animals through work or travel. We suspect that most of the patients acquired *Yersinia* through foodborne transmission. Two patients may have eaten the same cheese, although bacteriologic analysis of the cheese could not be performed. Serologic tests for yersiniosis and brucellosis were done for both patients; one was positive for *Brucella*, the other for *Yersinia*. Second specimens were both positive for *Yersinia* by the agglutination test and the new YOP ELISA test.

*Yersinia enterocolitica* infection is a protean disease (5). Gastrointestinal symptoms are the most frequent. In our series, many patients sought medical attention for persistent fever, night sweats, or secondary features of the disease (7); digestive symptoms were prominent in their history. At this stage of the disease, *Yersinia* could not be isolated from stools. As the common serologic tests (positive either with *Y. enterocolitica* or with *Brucella* antigens) were not useful, the absence of contact with animals infected with brucellosis was an indication of yersiniosis. Diagnosis could be confirmed by positive YOP ELISA.

The 1996 *Yersinia* epizootic in Auvergne preceded an increase in human cases in central France, where no cases of *Y. enterocolitica* O:9 had previously been detected. The epizootic demonstrates that such emerging disease can be predicted by veterinary surveillance data (9).

### Acknowledgments

The authors gratefully acknowledge the help of E. Carniel (Centre de Référence des Yersinia, Institut Pasteur, Paris).

### References

1. Douzal Y. Policy of prevention against bovine brucellosis. Actual results. Medecine et Maladies Infectieuses 1993;23(S):507-12.
2. Reynaud A, Delmas C, Vidon JM, Marty-Garrec M, Dubourdieu V. Presence of *Yersinia enterolitica* O:9 in fecal samples of *Brucella abortus* ruminants revealing atypical serological reactions against antigens. Medecine et Maladies Infectieuses 1993;23(S):516-9.
3. Kittelberger R, Reichel MP, Joyce MA, Skaak C. Serological crossreactivity between *Brucella abortus* and *Yersinia enterocolitica* O:9. III. Specificity of the in vitro antigen-specific gamma interferon test for bovine brucellosis diagnosis in experimentally *Yersinia enterocolitica* O:9-infected cattle. Vet Microbiol 1997;57(4):361-71.
4. Verhaegen J, Charlier J, Lemmens P, Delmee M, Van Noyen R, Verbist L, et al. Surveillance of human *Yersinia enterocolitica* infections in Belgium: 1967-1996. Clin Infect Dis 1998;27(1):59-64.
5. Bottone EJ. *Yersinia enterocolitica*: the charisma continues. Clin Microbiol Rev 1997;10(2):257-276.
6. Shorter NA, Thompson MD, Mooney DP, Modlin JF. Surgical aspects of an outbreak of *Yersinia* enterocolitis. Pediatr Surg Int 1998;13(1):2-5.
7. de Kleijn EM, van Lier HJ, van der Meer JW. Fever of unknown origin (FUO). II. Diagnostic procedures in a prospective multicenter study of 167 patients. The Netherlands FUO Study Group. Medicine 1997;76(6):401-14.
8. Benoit C, Guiyoule A, Carniel E. Serodiagnosis of human pathogenic *Yersinia.* Presse Med 1996;25:1627-30.
9. Tauxe RV. Emerging foodborne diseases: an evolving public health challenge. Emerg Infect Dis 1997;3(4):425-34.
